# Impact of SARS-CoV-2 Infection on the Outcomes of Trauma Patients at a Level I Trauma Center: An Ambispective Observational Study

**DOI:** 10.7759/cureus.82162

**Published:** 2025-04-13

**Authors:** Shrikant Manwatkar, Ramesh Vaidyanathan, Narendra Chaudhary, Abhinav Kumar, Pratyusha Priyadarshini, Dinesh Bagaria, Amit Gupta, Sushma Sagar, Subodh Kumar, Biplab Mishra

**Affiliations:** 1 Trauma Surgery and Critical Care, Command Hospital Air Force, Bengaluru, IND; 2 Trauma Surgery and Critical Care, All India Institute of Medical Sciences, New Delhi, IND

**Keywords:** covid-19, impact of sars-cov-2, sars cov-2, trauma, trauma mortality

## Abstract

Introduction: Trauma remained a leading cause of hospital admissions even during the COVID-19 pandemic. Trauma and surgical interventions are known to impair the patient’s immune function. Clinically, some asymptomatic COVID-19 patients experienced rapid deterioration following surgery. Surgeons and anesthesiologists need to be aware that acute lung injury caused by COVID-19 could be present preoperatively or may worsen postoperatively. Hence, an ambispective observational study was planned to assess the impact of severe acute respiratory syndrome coronavirus (SARS-CoV-2) infection on trauma patient outcomes.

Aims and objectives: This study aims to evaluate the impact of SARS-CoV-2 infection on the outcomes of trauma patients at a level I trauma center.

Materials and methods: This ambispective observational study was conducted at a level 1 trauma center and included patients admitted under the trauma surgery service in the COVID-19 facility. Their outcomes were compared with those of patients admitted to the non-COVID-19 facility from March 2020 to March 2022.

Results: A total of 2,017 patients were admitted under the Division of Trauma Surgery and Critical Care from March 2020 to March 2022. The mean duration of intercostal drainage (ICD) was significantly longer in SARS-CoV-2-positive trauma patients (7.03 ± 3.69 days) compared to SARS-CoV-2-negative trauma patients (5.28 ± 2.75). Acute respiratory distress syndrome (ARDS) was also more common among SARS-CoV-2-positive trauma patients. Additionally, these patients had a longer hospital stay. Notably, SARS-CoV-2-positive trauma patients who died had a significantly lower average injury severity score (ISS) compared to SARS-CoV-2-negative counterparts.

Discussion: Although the average ISS was lower and the average trauma and injury severity score (TRISS) was higher in SARS-CoV-2-positive trauma patients who died compared to SARS-CoV-2-negative trauma patients, overall mortality rates were comparable between the two groups.

Conclusion: Trauma patients with concomitant SARS-CoV-2 infection had a longer duration of ICD, along with an increased incidence of chest infections and ARDS. A greater proportion of SARS-CoV-2-positive trauma patients required ventilatory support. The mortality observed in SARS-CoV-2-positive trauma patients is likely attributed to the concomitant SARS-CoV-2 infection.

## Introduction

In December 2019, Wuhan, China, reported a cluster of pneumonia cases of unknown origin. On December 31, 2019, the WHO identified the causative agent as a novel strain of coronavirus, initially termed 2019-nCoV [1]. The International Committee on Taxonomy of Viruses later renamed it severe acute respiratory syndrome coronavirus 2 (SARS-CoV-2). The infection rapidly spread from China to many countries worldwide and was declared a public health emergency of international concern on January 30, 2020 [2].

COVID-19 patients may be asymptomatic or present with symptoms of respiratory tract infection and may show features of viral pneumonia on chest CT scans. In emergency or trauma settings, assessing these symptoms can be particularly challenging. Trauma patients may present as either symptomatic or asymptomatic carriers of COVID-19. Diagnosing COVID-19 infection in such patients requires careful evaluation and remains a clinical challenge [2].

Globally, stringent quarantine and control measures were implemented to control the spread of SARS-CoV-2. However, severe trauma remained a leading cause of hospital admission even during this period. The trauma patient population was heterogeneous with respect to infection status, including previously healthy individuals, suspected or confirmed cases, close contacts of infected individuals, and asymptomatic carriers. As a result, delivering optimal trauma care while preventing further spread of the virus posed a major challenge.

Trauma and surgical interventions can impair a patient’s immune function. Clinically, some asymptomatic COVID-19 patients experienced rapid deterioration following surgery. Surgeons and anesthesiologists need to be aware that acute lung injury caused by COVID-19 may be present preoperatively or may worsen postoperatively. In postoperative trauma patients presenting with fever, it is essential to differentiate between COVID-19 and complications related to trauma or surgery. Similarly, postoperative dyspnea and hypoxia must be carefully evaluated to distinguish them from other complications, such as pulmonary embolism [3]. COVID-19 infection in trauma patients has posed a challenge and may have altered the natural course of trauma recovery [4].

Therefore, to assess the impact of SARS-CoV-2 infection on trauma patients, a combined retrospective and prospective observational study was planned.

## Materials and methods

This study aims to evaluate the impact of SARS-CoV-2 infection on the outcomes of trauma patients at a level I trauma center. The objectives were to compare the outcomes between trauma patients with and without concomitant SARS-CoV-2 infection, admitted under the Division of Trauma Surgery and Critical Care, with respect to the following parameters: duration of ICD, need for ventilatory support, incidence of chest infections, ARDS, pulmonary thromboembolism, myocardial infarction (MI), stroke, length of intensive care unit (ICU) stay, length of hospital stay, and mortality.

This ambispective observational study was conducted at Jai Prakash Narayan, Apex Trauma Center, AIIMS, New Delhi, a level 1 trauma center, during the COVID-19 pandemic, from March 2020 to March 2022. The study was approved by the institutional ethics committee. All patients admitted under the Division of Trauma Surgery and Critical Care, with or without concomitant SARS-CoV-2 infection, were included in the study. Group A comprised patients admitted under trauma surgery in the COVID-19 facility with concomitant SARS-CoV-2 infection, while Group B included trauma patients admitted to the non-COVID-19 facility without concomitant SARS-CoV-2 infection. There were no specific exclusion criteria.

All the data collected were recorded in a Microsoft Excel spreadsheet (Microsoft Corp., Redmond, WA, USA). The data were then coded and analyzed using IBM SPSS Statistics for Windows, Version 23.0 (Released 2015; IBM Corp., Armonk, NY, USA). Descriptive statistics were presented as means and standard deviations or medians and interquartile ranges (IQRs) for continuous variables and as frequencies and percentages for categorical variables. Group comparisons were performed using the Wilcoxon test, chi-squared test, and Fisher’s exact test. A p-value of <0.05 was considered statistically significant.

## Results

**Figure 1:**
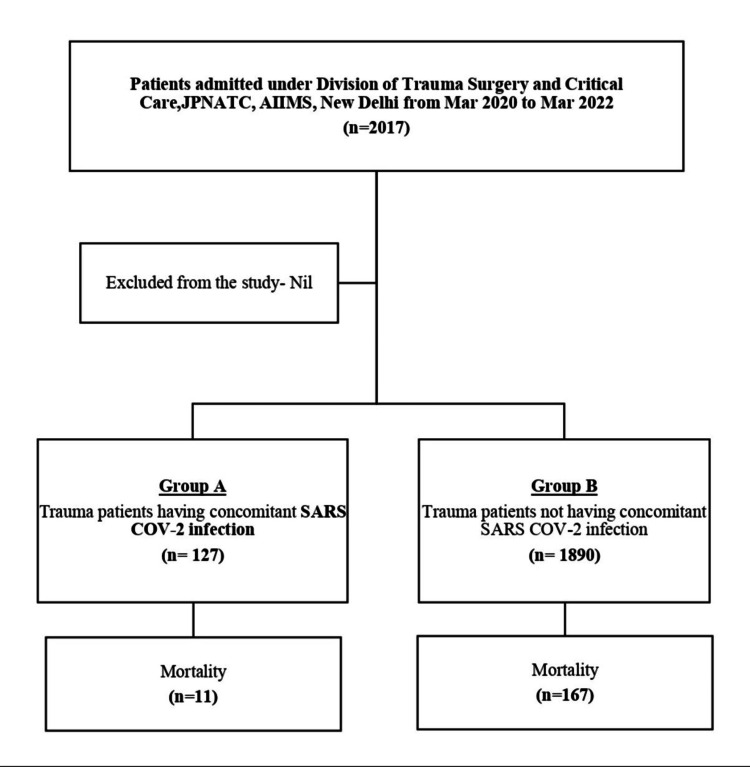
CONSORT diagram

A total of 2,017 patients were admitted under the Division of Trauma Surgery and Critical Care from March 2020 to March 2022. Of them, 127 trauma patients had concomitant SARS-CoV-2 infection (Group A) and were admitted to a dedicated COVID-19 facility, while 1,890 patients without concomitant SARS-CoV-2 infection (Group B) were admitted to a non-COVID-19 facility (Figure 1, Table 1).

**Table 1:** Demographics and type of management of patients p-value is significant at p < 0.05. *Wilcoxon-Mann-Whitney U test. **Chi-squared test.

	SARS-CoV-2 infection		Test value	p-value
	Yes (n = 127)	No (n = 1890)		
Age, mean ± SD	33.46 ± 13.34	33.91 ± 14.63	117,775.500	0.724*
Male	117 (92.1%)	1655 (87.6%)	2.319	0.128**
Female	10 (7.9%)	235 (12.4%)		
Non-operative management	38 (29.9%)	662 (35.0%)	3.128	0.209**
Non-operative intervention	24 (18.9%)	260 (13.8%)		
Operative management	65 (51.2%)	968 (51.2%)		

The mean age of patients in Group A was 33.46 ± 13.34 years, while in Group B, it was 33.91 ± 14.63 years. Both groups were comparable in terms of age distribution (p = 0.724).

In Group A, 92.1% (n = 117) were male and 7.9% (n = 10) were female patients. In Group B, 87.6% (n = 1655) were male and 12.4% (n = 235) were female patients. Both groups were comparable in terms of gender distribution (p = 0.128).

In Group A, 38 patients (29.9%) were managed with non-operative management, 24 patients (18.9%) with non-operative intervention, and 65 patients (51.2%) with operative management. In Group B, 662 patients (35.0%) underwent non-operative management, 260 patients (13.8%) received non-operative intervention, and 968 patients (51.2%) underwent operative management.

**Figure 2:**
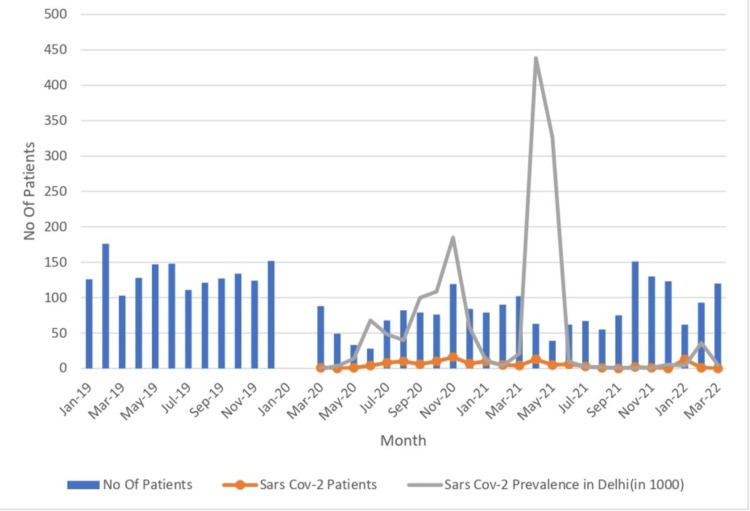
Month-wise patient distribution compared with the 2019 (pre-COVID-19) data Month-wise patient distribution from March 2020 to March 2022 (study period) compared with the data of the previous year, i.e., January 2019 to December 2019 (pre-COVID period). That is why there is no data for January 2020 and February 2020.

**Figure 3:**
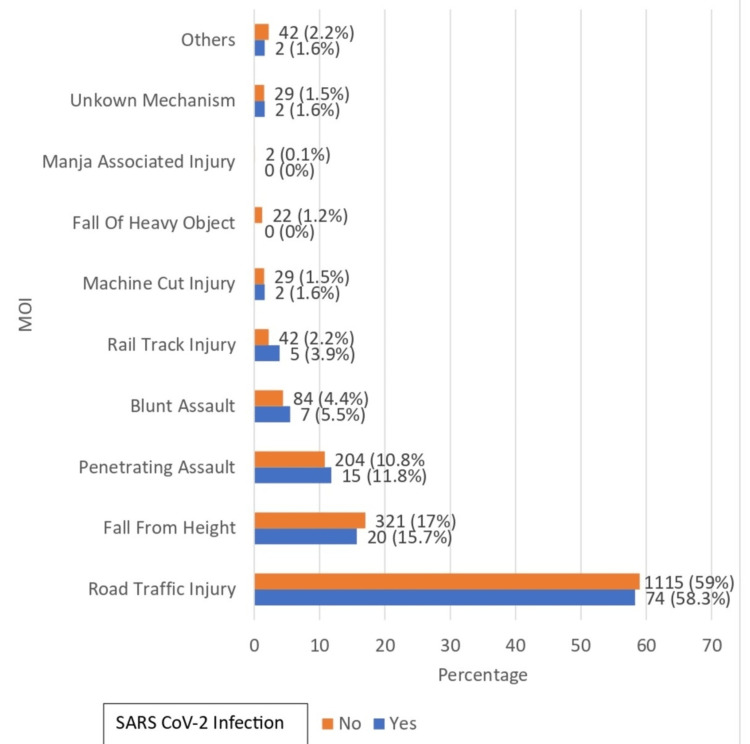
Association between SARS-COV-2 infection and mode of injury (MOI) Manja is a thread coated with finely crushed glass, traditionally used for flying kites in India. However, it has been associated with injuries, particularly when left on roads, where it can cause neck injuries in two-wheeler riders.

There was no statistically significant difference in the type of management between the two groups (p = 0.209) (Figure 2 and Figure 3).

Road traffic injury (RTI) was the most common mode of injury (MOI) observed in 58.3% (n = 74) of patients in Group A and 59% (n = 115) in Group B, followed by falls from height and penetrating assault. Both groups were comparable in terms of MOI distribution (p = 0.9) (Table 2).

**Table 2:** Comparison between trauma patients with and without SARS-CoV-2 infection, with respect to injury severity score (ISS), new injury severity score (NISS), trauma and injury severity score (TRISS), intercostal drain (ICD), duration of ICD, chest infection, acute respiratory distress syndrome (ARDS), ventilatory support, duration of ventilatory support, pulmonary thromboembolism, stroke, myocardial infarction (MI), hospital stay, intensive care unit (ICU) admission, ICU stay, and mortality p-value is significant at p < 0.05. *Wilcoxon-Mann-Whitney U test. **Chi-squared test. ***Fisher’s exact test.

Injury score	SARS-CoV-2 infection		Test value	p-value
	Yes (n = 127; Group A)	No (n = 1,890; Group B)		
ISS	16.62 ± 9.39	17.20 ± 10.10	116,623.000	0.593*
NISS	14.63 ± 8.51	14.48 ± 8.89	122,567.000	0.687*
TRISS	97.75 ± 6.32	96.62 ± 10.39	118,653.000	0.979*
ICD	32 (25.2%)	438 (23.2%)	0.272	0.602**
Duration of ICD (days)	7.03 ± 3.69	5.28 ± 2.75	9,386.500	0.001*
Chest infection	18 (14.2%)	26 (1.4%)	91.337	<0.001***
ARDS	27 (21.3%)	36 (1.9%)	147.332	<0.001***
Ventilatory support	30 (23.6%)	288 (15.2%)	6.299	0.012**
Duration of ventilatory support (days)	12.00 ± 10.97	9.24 ± 11.63	4,921.000	0.209*
Pulmonary thromboembolism	3 (2.4%)	3 (0.2%)	19.482	0.004***
Stroke	2 (1.6%)	0 (0.0%)	29.793	0.004***
MI	2 (1.6%)	1 (0.1%)	18.559	0.011***
Hospital stay (days)	16.09 ± 20.72	12.09 ± 14.86	143,784.500	<0.001*
ICU admission	87 (68.5%)	319 (16.9%)	197.279	<0.001**
ICU stay (days)	10.03 ± 7.91	11.10 ± 12.13	13,922.500	0.962*
Mortality	11 (8.7%)	167 (8.8%)	0.005	0.946**

The mean ISS in Group A was 16.62 ± 9.39, while in Group B, it was 17.20 ± 10.10, with no significant difference between the groups (p = 0.593).

The mean NISS in Group A was 14.63 ± 8.51, compared to 14.48 ± 8.89 in Group B, with no significant difference between the groups (p = 0.687).

The mean TRISS in Group A was 97.75 ± 6.32, while in Group B, it was 96.62 ± 10.39, with no significant difference between the groups (p = 0.979).

In Group A, 25.2% (n = 32) of patients had ICD, compared to 23.2% (n = 438) in Group B, with no significant difference between the groups (p = 0.602).

The mean duration of ICD was significantly longer in Group A (7.03 ± 3.69 days) compared to Group B (5.28 ± 2.75 days) (p = 0.001), indicating a greater need for prolonged chest drainage in SARS-CoV-2-positive trauma patients.

In Group A, 14.2% (n = 18) of patients developed chest infections, compared to 1.4% (n = 26) in Group B. Chest infections were significantly more common in SARS-CoV-2-positive trauma patients (p < 0.001).

Similarly, 21.3% (n = 27) of patients in Group A developed ARDS, compared to 1.9% (n = 36) in Group B, indicating a significantly higher incidence of ARDS among SARS-CoV-2-positive trauma patients (p < 0.001).

Mechanical ventilatory support was required in 23.6% (n = 30) of patients in Group A, compared to 15.2% (n = 288) in Group B. The difference was statistically significant (p = 0.012), with a greater proportion of SARS-CoV-2-positive trauma patients requiring mechanical ventilation.

The mean duration of ventilation required in Group A was 12.00 ± 10.97 days, while in Group B, it was 9.24 ± 11.63 days. There was no significant difference in the duration of mechanical ventilation in both groups (p = 0.209). However, a significant difference was observed in the duration of ventilatory support and mortality (p = 0.001) (Table 3).

**Table 3:** Association between duration of ventilatory support and mortality. p-value is significant at p < 0.05.

Duration of ventilatory support (n=318)	Mortality		Wilcoxon-Mann-Whitney U test	
	Yes	No	Test value	p-value
Mean ± SD	9.45 ± 14.45	9.56 ± 7.46	9,816.000	0.001

The average hospital stay was 16.09 ± 20.72 days in Group A and 12.09 ± 14.86 days in Group B. SARS-CoV-2-positive trauma patients (Group A) had a significantly longer hospital stay (p < 0.001).

In Group A, 68.5% (n = 87) of patients required ICU admission, compared to 16.9% (n = 319) in Group B. A significantly higher proportion of SARS-CoV-2-positive trauma patients (Group A) required ICU admission (p < 0.001).

The average ICU stay was 10.03 ± 7.91 days in Group A and 11.10 ± 12.13 days in Group B. There was no significant difference found between the groups (p = 0.962).

The total mortality was 11 (8.7%) patients in Group A and 167 (8.8%) patients in Group B. There was no significant difference in the number of deaths between the groups (p = 0.946) (Table 3).

**Table 4:** Comparisons of mortality in trauma patients with and without SARS CoV-2 infection, with respect to injury severity score (ISS) and trauma and injury severity score (TRISS) p-value is significant at p < 0.05. "Yes" is the presence of SARS-CoV-2 infection, while "No" is the absence of SARS-CoV-2 infection. *Wilcoxon-Mann-Whitney U test.

Mortality (N = 178)				
	SARS-CoV-2 infection		Test value	p-value
	Yes (n = 11)	No (n = 167)		
ISS	17.64 ± 10.61	26.20 ± 11.83	240,361.000	0.032*
TRISS	92.63 ± 19.11	82.38 ± 25.42	63,157.000	0.017*

The average ISS in patients with mortality was 17.64 ± 10.61 in Group A and 26.20 ± 11.83 in Group B. SARS-CoV-2-positive trauma patients (Group A) had a significantly lower average ISS score (p = 0.032).

The average TRISS was 92.63 ± 19.11 in patients with mortality in Group A and 82.38 ± 25.42 in Group B. The average TRISS was significantly higher in SARS-CoV-2-positive trauma patients (Group A) (p = 0.017).

## Discussion

The Jay Prakash Narayan Apex Trauma Center, AIIMS, New Delhi, is a level I trauma center serving patients from the Delhi NCR and surrounding states [5]. Approximately 1,500 patients are admitted annually under the Division of Trauma Surgery and Critical Care. However, due to the nationwide lockdown and the impact of the COVID-19 pandemic, the number of trauma patients admitted decreased to approximately 1,000 a year. This decline in trauma cases aligns with the findings from studies by D’Angelo et al. [6] and Roshanaei et al. [7], which reported a reduction in trauma admissions by 65.55% and 44%, respectively, compared to pre-pandemic levels.

A total of 2,017 patients were admitted during the study period from March 2020 to March 2022. Of them, 127 patients were concomitantly infected with SARS-CoV-2.

The most common age group in Group A and Group B was 21-30 years. The age distribution among COVID-19 and non-COVID-19 trauma patients was comparable. Among the 127 SARS-CoV-2-positive trauma patients (Group A), 117 were male and 10 were female patients. In contrast, of the 1,890 SARS-CoV-2-negative trauma patients (Group B), 1,655 were male and 235 were female patients. The gender distribution was comparable in both groups. These findings were consistent with studies by Jain et al. [8] and Onyemaechi [9].

The most common MOI in Group A (58.3%) and Group B (59%) was RTI, consistent with the findings of Jain et al. [8]. In Group A, 15 (11.8%) patients presented with penetrating assault, while seven (5.5%) had blunt assault injuries. In Group B, 204 (10.8%) patients presented with penetrating assault, while 84 (4.4%) had blunt assault. Although the proportion of assault cases was slightly higher in Group B, the difference between the groups was not statistically significant.

Thirty-eight patients (29.9%) in Group A and 662 (35.0%) in Group B were treated with non-operative management. Non-operative intervention was performed in 24 patients (18.9%) in Group A and 260 patients (13.8%) in Group B. Operative management was undertaken in 65 patients (51.2%) in Group A and 968 patients (51.2%) in Group B. Overall, patient management was comparable between the two groups.

The mean ISS was 16.62 ± 9.39 in Group A and 17.20 ± 10.10 in Group B. These findings are consistent with a study by Narayanan et al., who reported a mean ISS of 15.63 ± 8.85 [10]. The mean NISS in Group A was 14.63 ± 8.51, compared to 14.48 ± 8.89 in Group B. The mean TRISS in Group A was 97.75 ± 6.32, whereas in Group B, it was 96.62 ± 10.39. The ISS values were comparable in both groups.

In Group A, 32 (25.2%) patients had an ICD, compared to 438 (23.2%) patients in Group B. The mean duration of ICD was 7.03 ± 3.69 days in Group A and 5.28 ± 2.75 in Group B. This was consistent with the findings of Narayanan et al., who reported a mean ICD duration of 6.94 ± 4.21 days, with a median of six days [10]. Notably, the duration of ICD use was longer in SARS-CoV-2-positive trauma patients (Group A).

Eighteen (14.2%) patients in Group A developed chest infections, compared to 26 (1.4%) in Group B. This finding is consistent with a study by Yeates et al., reporting a higher incidence of pneumonia in COVID-19 patients compared to non-COVID-19 trauma patients [11]. The rate of chest infections was significantly higher in SARS-CoV-2-positive trauma patients (Group A). However, the chest infection rate in our study is slightly lower than that reported in a similar study by Abe et al., where the pneumonia rate in trauma patients was 3.2% [12].

In Group A, 27 (21.3%) patients developed ARDS, compared to 36 (1.9%) in Group B. The incidence of ARDS in SARS-CoV-2-negative trauma patients (Group B) was lower than that reported in a systematic review by Pfeifer et al., which found a median ARDS incidence of 8.4% [4]. The higher incidence of ARDS reported in that review may be due to the fact that it included only ICU-admitted patients, whereas our study considered ARDS cases from both the ICU and general wards. The incidence of ARDS was significantly higher in SARS-CoV-2-positive trauma patients (Group A).

Thirty (23.6%) patients in Group A and 288 (15.2%) in Group B required ventilatory support. A higher proportion of SARS-CoV-2-positive trauma patients (Group A) required ventilatory assistance. The average duration of ventilatory support was 12.00 ± 10.97 days in Group A and 9.24 ± 11.63 days in Group B. These findings are consistent with a study by Yeates et al., which reported no significant difference in ventilator days between COVID-19 and non-COVID-19 trauma patients [11].

Pulmonary thromboembolism occurred in three patients (2.4%) in Group A and in three patients (0.2%) in Group B. This aligns with similar studies by Shuster et al. [13] and Bahloul et al. [14], who reported pulmonary thromboembolism rates in trauma patients ranging from 0.35% to 24%. In Group A, two patients (1.6%) developed stroke, while no cases of stroke were reported in Group B. Additionally, two patients (1.6%) in Group A experienced MI, compared to one patient (0.1%) in Group B. The incidence of pulmonary thromboembolism, stroke, and MI was notably higher in SARS-CoV-2-positive trauma patients (Group A). However, these results should be interpreted with caution, as the small sample size limits generalizability to the broader population.

The average hospital stay in Group A was 16.09 ± 20.72 days, compared to 12.09 ± 14.86 days in Group B. This finding is consistent with a study by Yeates et al., who reported that COVID-19 patients had a longer mean length of stay (LOS) than non-COVID-19 trauma patients (7.47 vs. 3.28 days, p < 0.001) [11]. However, the mean hospital stay in that study was shorter than in the present study, possibly due to lower ISS scores among the patients included in the study by Yeates et al. [11]. ICU admission was required for 87 patients (68.5%) in Group A, compared to 319 patients (16.9%) in Group B. Both hospital stay duration and ICU admission rates were significantly higher in SARS-CoV-2-positive trauma patients (Group A). The average ICU stay was 10.03 ± 7.91 days in Group A and 11.10 ± 12.13 days in Group B. The ICU stay was slightly shorter in SARS-CoV-2-positive patients (Group A), which may be attributed to lower ISS scores in this group. These findings are consistent with a study by Böhmert et al. (2014), who reported an average ICU stay of 11.5 days in trauma patients [15].

Eleven patients (8.7%) in Group A and 167 patients (8.8%) in Group B died. There was no significant difference in mortality between COVID-19 and non-COVID-19 trauma patients. However, a similar study by Yeates et al. reported higher mortality in COVID-19 trauma patients compared to their non-COVID-19 counterparts [11].

The average ISS among patients who died in Group A was 17.64 ± 10.61, compared to 26.20 ± 11.83 in Group B. The average TRISS score was 92.63 ± 19.11 in deceased patients from Group A and 82.38 ± 25.42 in Group B. Although the average ISS was lower and the TRISS score was greater, indicating a higher probability of survival, in SARS-CoV-2-positive trauma patients (Group A) compared to SARS-CoV-2-negative patients (Group B), the overall mortality rates in both groups were comparable. This suggests that mortality in SARS-CoV-2-positive trauma patients may be influenced by the presence of concomitant SARS-CoV-2 infection.

Limitations

This was a single-center study with a mixed retrospective and prospective observational design. Additionally, the number of SARS-CoV-2-positive trauma patients included was relatively small (n = 127), which may limit the generalizability of the findings.

## Conclusions

This ambispective observational study, conducted at a level I trauma center, found that trauma patients with concomitant SARS-CoV-2 infection had a longer duration of ICD and a higher incidence of chest infections and ARDS. A greater proportion of SARS-CoV-2-positive trauma patients required ventilatory support and experienced complications such as pulmonary thromboembolism, stroke, and MI. These patients also had longer hospital stays and higher ICU admission rates. Although overall mortality was comparable between SARS-CoV-2-positive and SARS-CoV-2-negative trauma patients, the mortality observed in SARS-CoV-2-positive patients may be attributed to the concomitant SARS-CoV-2 infection.
